# Supplementary material for: Polyketide synthase gene clusters in the ericoid mycorrhizal fungus Oidiodendron maius: genome-wide prediction, expression profiling, and functional characterization of knockout mutants
Source: Front Plant Sci. 2026 Sep 16;17:1924430. doi: 10.3389/fpls.2026.1924430 (PMC13623903; doi:10.3389/fpls.2026.1924430)
Supplement: Supplementary file 1 [file SupplementaryFile1.zip › Supplementary Materials/Supplementary Folder 1.zip!/Oidma1_AssemblyScaffolds_Repeatmasked/knownclusterblast/cluster13/jgi.p_Oidma1_51169_mibig_hits.html]

| MIBiG Protein | Description | MIBiG Cluster | MiBiG Product | % ID | % Coverage | BLAST Score | E-value |
| --- | --- | --- | --- | --- | --- | --- | --- |
| BAV32142.1 | putative\_alpha-ketoglutarate-dependent\_taurine\_dioxygenase\_protein | BGC0001373 | Sordarin\_biosynthetic\_gene\_cluster | 56 | 92.6509186352 | 380 | 2e-105 |
| KGO40485.1 | Taurine\_catabolism\_dioxygenase\_TauD/TfdA | BGC0001205 | Communesin\_biosynthetic\_gene\_cluster | 35 | 85.0393700787 | 176 | 7.4e-44 |
| AGC09525.1 | dioxygenase | BGC0001183 | Lobophorin\_biosynthetic\_gene\_cluster | 38 | 71.9160104987 | 172 | 8.2e-43 |
| ctg1\_orf6 |  | BGC0000687 | Phyllocladan-16alpha-ol\_biosynthetic\_gene\_cluster | 49 | 20.9973753281 | 76 | 6.1e-14 |
